# Diagnostic Difficulties in Discriminating Autism Spectrum Disorder in an Adult With Periodic Psychosis Versus Schizophrenia Spectrum Condition: An Insight From Psychological Testing

**DOI:** 10.7759/cureus.21887

**Published:** 2022-02-03

**Authors:** Siara M Clos, Faiz Kidwai, Susan Sperry, Luba Leontieva

**Affiliations:** 1 Psychiatry, State University of New York Upstate Medical University, Syracuse, USA; 2 Psychology, State University of New York Upstate Medical University, Syracuse, USA

**Keywords:** psychological testing, psychosis, schizoaffective disorder, schizophrenia spectrum disorder (sdd), autism spectrum disorder (asd)

## Abstract

It has long been recognized that the biological underpinnings of autism spectrum disorder (ASD) and schizophrenia spectrum disorder (SSD) may share a common basis; however, the two conditions remain separate in the Diagnostic and Statistical Manual of Mental Disorders, 5th Edition (DSM-5) due to a few distinguishing characteristics. Both disorders are characterized by cognitive and social deficits and have been presumed to be linked to multiple genes. We describe a 46-year-old male who presented atypically with three previous and one current episode of schizoaffective-like symptoms. We describe his previous inpatient admissions, current inpatient course, psychological test results, and treatment. The patient initially presented with schizoaffective disorder, but with a thorough interview, collateral information review, and psychological evaluation, it was determined that he instead was presenting with a previously undiagnosed case of ASD with brief psychosis when under stress. This case serves as an example of an atypical presentation of ASD which can be mistaken for schizoaffective disorder. It is important to establish the correct diagnosis, as the subsequent treatment and management of the patient’s problems will depend on it. In such a patient, a low dose of atypical antipsychotic medication with serotonergic properties and psychotherapy would be the treatment of choice.

## Introduction

Autism spectrum disorder (ASD) and schizophrenia spectrum disorder (SSD) may be far more interconnected than previously believed and dual diagnoses may occur more frequently than previous literature suggests. They remain as separate diagnoses in the Diagnostic and Statistical Manual of Mental Disorders, 5th Edition (DSM-5). ASD is characterized by social and communication dysfunction as well as restrictive and repetitive behavior, whereas SSD is characterized by delusions, hallucinations, disorganized thinking or speech, disorganized motor behavior, and negative symptoms. SSD includes schizophrenia, schizoaffective disorder, schizophreniform disorder, and schizotypal personality disorder. It is theorized that multiple genes are involved in both disorders. A common genetic underpinning could be rooted in an imbalance in the ratio of excitatory to inhibitory neuronal activity. Responsible genes are proposed to be those that code for synaptic proteins or GABAergic/glutamatergic receptors.

Per the DSM-5 as aforementioned, criteria for ASD includes both social and communicative deficiencies and repetitive or odd, intense behavior. To meet schizophrenia criteria, one must exhibit at least two of: delusions, hallucinations, disorganized thinking or speech, disorganized motor behavior, or negative symptoms for six months. Schizoaffective disorder is characterized by having symptoms that meet the criteria for a mood disorder for most of the illness and having symptoms that meet schizophrenia criteria for at least two weeks without mood episodes during the illness. Schizophreniform disorder is characterized by meeting schizophrenia criteria for one to six months. Schizotypal personality disorder is characterized by a pervasive pattern of abnormal perceptions and cognition, eccentricity, and lack of close relationships. Childhood-onset schizophrenia (COS) is characterized by symptoms of schizophrenia before the age of 13. A longitudinal study examining COS discovered that 50% of subjects had an underlying developmental disorder that preceded the onset of psychotic symptoms and 30% had ASD. ASD can include unusual perceptual experiences, odd thinking, preoccupations, and odd speech as in SSD. Both disorders are characterized by a lack of ability to mentalize. The negative symptoms of schizophrenia may be closely linked with the social impairments of ASD. The disorganized behaviors of schizophrenia may be closely linked with the repetitive movements and expressions of ASD. Both ASD and SSD are associated with social inadequacies, blunted affect, lack of facial expression, lack of speech differentiation, lack of gestures, lack of eye contact, odd language, and concrete or obsessive thought processes [1].

It has been identified that SSD and ASD are neurodevelopmental disorders in nature. SSD is associated with abnormal neural development in the dorsolateral and ventrolateral prefrontal regions of the brain while ASD is associated with abnormal neural development in the prefrontal and somatosensory areas of the brain. Both disorders are associated with changes in epigenetics and copy number variants (CNVs). They are both associated with increased paternal age as well as maternal infection or autoimmunity while in utero [2]. 

Many mechanisms have been proposed to account for the excitatory/inhibitory imbalance that characterizes both disorders. Deletions in the neurexin-1α gene, a synapse organizer, have been associated with ASD and schizophrenia. Astrocyte abnormalities have been noted in both disorders as well. Myelin and oligodendrocyte-related gene abnormalities, decreased numbers of oligodendrocytes, and defective maturation of cells have been detected in schizophrenia. High levels of active microglial cells have been detected in ASD [3]. 

Symptoms of ASD often overlap with symptoms of schizophrenia; however, the largest point of differentiation lies in positive symptoms. Psychological testing on ASD patients often centers on repetitive behavior and odd language patterns. Psychological testing on schizophrenic patients often detects more delusions and hallucinations. When the presentation has aspects of both ASD and SSD and when attempting to differentiate between disorders, it is important to consider whether symptoms of the patient’s predominant disorder are causing an elevation in measures designed to detect the other disorder [1].

## Case presentation

The patient is a 46-year-old male with a past psychiatric history of depression, social anxiety, psychosis, obsessive-compulsive disorder (OCD), and post-traumatic stress disorder (PTSD), and a past medical history of hypertension and hypercholesterolemia. He had three previous psychiatric hospitalizations. He presented to an emergency department (ED) with auditory and visual hallucinations that had worsened over two months.

He described the auditory hallucinations as "telepathy," like hearing the thoughts of others. The voices commanded him to perform tasks such as to check his email or to run a specific number of laps at the gym. He reported that if he ignored the commands, the voices would begin making belittling comments. The voices never commanded him to perform violent actions. He maintained that if they had, he would have ignored them. The voices had begun interfering with his daily life. He felt the voices were very authentic and certain circumstances made him feel as though he was the target of a conspiracy. 

He additionally reported mild anxiety and depression associated with the hallucinations, but these symptoms were manageable and not overly bothersome. The voices made it difficult to initiate sleep and thus he often felt sleep deprived. He stated he had a long history of sleep disturbance.

History of present illness

The patient's most recent episode of psychosis began after he was the victim of identity theft. He reported having auditory and visual hallucinations as well as a "drained" mood at that time. He also became paranoid and mistrustful of others, even those uninvolved in the theft. This led him to abruptly resign from his occupation and liquidate his 401K. Shortly thereafter, he was seen at a Comprehensive Psychiatric Emergency Program (CPEP). This was his fourth recurrence of psychosis; therefore, his previously prescribed Aripiprazole dose was increased to 10mg daily. His antidepressant was additionally switched from Fluoxetine 20mg daily to Bupropion after complaints of impotence and he was started on Clonidine 0.2mg nightly due to hypertension. Shortly after discharge, he reported 50 hours of insomnia. This prompted an ED visit where a one-time dose of Aripiprazole 5mg was administered. Due to previous episodes of akathisia on Aripiprazole, Lurasidone 20mg daily was initiated the following day. He was additionally tapered off Bupropion and restarted on Fluoxetine 20mg daily.

He was subsequently admitted to a psychiatric inpatient unit where he continued to report visual and auditory hallucinations leading to insomnia. One visual hallucination included a holographic image of President Donald Trump eating potato chips next to him. Auditory hallucinations included voices that he felt came from presidents, celebrities, governors, family members, and coworkers. His auditory and visual hallucinations often occurred independently, but he felt they were connected. He felt the voices could read his thoughts and predict somatic sensations. For example, the voices occasionally told him that his ankle or stomach hurt. He would soon thereafter experience those symptoms. The voices also had access to his memories. He cited an example where he could not remember his room number, so the voices told him. He also said the "CEO of Google" often accurately told him how many emails he had. He recognized the voices as fictitious, but he felt as though he was the target of a governmental conspiracy for leaving his last job without giving notice. 

Past psychiatric history

The patient saw a psychiatrist for the first time at the age of 19 after suffering accusations of homosexuality from a peer in high school. He became obsessed over the accusations, often experiencing intrusive thoughts of men coming onto him. This led his psychiatrist to diagnose him with OCD. 

His first episode of psychosis requiring psychiatric hospitalization began during his undergraduate study a few years later. Paranoia led him to resign from his part-time student occupation as he felt the company’s president disliked him based on frequently furrowing his eyebrows. This was the first time he had resigned from an occupation. Since then, he had functioned well, was able to receive a graduate degree, and was gainfully employed.

The patient's second episode of psychosis occurred at the age of 41. This included his first auditory hallucination. He reported an episode of trismus and intermittent muscle contraction followed a few days later by voices in his head. He was not on any psychiatric medication at that time. Over a few weeks to months, his symptoms drove him to resign from his occupation for the second time and drive to a Western state “to clear his head.” He reported hearing President Barack Obama's voice on the drive back telling him to pull over and leave his car. He was found confused on the side of the road by another driver who called an ambulance. The patient reported it was the Whitehouse Rescue Squad who brought him to a psychiatric facility. He felt that the communication by President Obama and the transportation to the hospital by the Whitehouse Rescue Squad were connected. He was hospitalized for the second time, diagnosed with psychosis and a possible bipolar condition, and placed on Aripiprazole. He experienced symptom relief in two weeks. He had begun new work as a lab technician and after a few months without hallucinations, he decided to discontinue the medication. 

Two years later at the age of 43, he began experiencing some subjective short-term memory loss, tension headaches, and vertigo prompting him to see a neurologist. He had a workup including an electroencephalogram (EEG), magnetic resonance angiography (MRA) of the brain, carotid doppler, sleep study, and laboratory studies which were all normal. He was prescribed Meclizine, Ondansetron, and Melatonin. He reported worsening dizziness as well as brain fog and a pressure-like sensation in his head, prompting him to present to an ED where he had a negative computed tomography (CT) of the brain and CT angiogram (CTA) of the brain and neck vessels. He was discharged with Meclizine, Fluticasone nasal spray, and Hydrochlorothiazide.

Soon after his ED visit, his auditory and visual hallucinations resumed. He believed this was due to the MRI “jarring something loose” or from a reaction to the contrast dye. To “clear his head” again, he decided to drive to another city. He reportedly got lost in the middle of the night, got a flat tire, walked to the nearest rest station, and called the police. He then underwent his third psychiatric admission. He was treated with Haloperidol and Ziprasidone without relief. He was then given injectable Aripiprazole 400mg. He reported his auditory and visual hallucinations lasted three months following the admission. Additionally, as a side effect of the Aripiprazole, he experienced hand tremors. This caused him to resign from his occupation without giving notice for the third time, which he ultimately felt guilty about. He was reinitiated on oral Aripiprazole 10mg daily and was prescribed Fluoxetine 20mg daily for anxiety and depression. He was then able to find work as a lab technician again. In 2019 at the age of 44, his Aripiprazole dose was decreased to 5mg daily and in 2020 at the age of 45, to 2.5mg daily which decreased the hand tremors.

Family history

His brother had an unspecified mental illness; his father had a gambling disorder.

Social history

He reported a positive childhood with supportive parents and no developmental concerns. There was no in utero exposure and his delivery was uncomplicated. He had no childhood illnesses, surgeries, or hospitalizations. All his milestones were met. He received his bachelor’s degree in communications and worked two years toward a master’s degree. He later decided on a career change and received his associate degree in medical lab technology. He had his most recent job for two-and-a-half years in a lab until spontaneously quitting. He never required any special accommodations and was never diagnosed with a learning disability. He lived alone and was not involved in a romantic relationship. He tried online dating in the past to no avail. He reported parental support but no relationship with his brother, not many friends, and no children. He reported occasionally going out to eat with coworkers in the past but had not been doing this in recent years. He had never used drugs, tobacco, or alcohol. Regarding trauma, the patient was verbally abused and witnessed his brother being physically abused by a neighbor during his childhood.

Current hospital course

Throughout his time on the unit, he maintained good hygiene, was cooperative, and maintained good eye contact. He did not exhibit psychomotor agitation, psychomotor retardation, or restlessness. His speech was normal in quantity, rate, and volume. His mood was reportedly “good” throughout his stay. His affect was euthymic, stable, appropriate, and congruent with his stated mood. He did appear blunted but with full range. His thought process remained linear, coherent, and goal directed. He never experienced delusions but did report auditory and visual hallucinations while being on the unit. He did not appear internally preoccupied. He maintained good insight and judgement. His reported psychopathology did not correspond to his behavior; he was calm and composed yet reported voices telling him what to do.

A comprehensive metabolic panel was run on the patient while he was on the unit. It was significant only for slightly elevated low-density lipoprotein (LDL) cholesterol at 103. Thyroid stimulating hormone (TSH), folate, and vitamin B1, B6, and B12 levels were all within normal limits. His syphilis IgG/IgM screen as well as human immunodeficiency virus antigen-antibody combination screen were negative. A heavy metal panel was also negative.

His Lurasidone was titrated up to 40mg daily. Quetiapine 50mg nightly was begun for sleep to good effect. Benztropine 1mg twice daily was initiated to protect against extrapyramidal symptoms. Due to hypertensive spikes halfway through the admission, Losartan 50mg twice daily was initiated. Neurologic etiology including seizure was ruled out as the cause of his psychiatric symptoms by a neurology team.

The patient was referred for psychological testing with a consulting psychologist who completed a clinical interview using the Minnesota Multiphasic Personality Inventory - Second Edition (MMPI-2) and the Thematic Apperception Test (TAT). On the MMPI-2, the patient’s most elevated clinical scale was Scale 6 (Pa; T = 83), suggesting strong interpersonal sensitivity, feeling misunderstood and resentful, feeling as though the world was a threatening place, and the presence of psychotic symptoms and delusions. The next highest elevation on his profile was Scale 2 (D; T = 74), which reflected endorsement of items suggesting that he may have been feeling depressed, lacking in energy to cope, uninterested in things around him, inferior, uncomfortable in social situations, lacking in self-confidence, socially avoidant, and emotionally immobilized. Patterns of social isolation, estrangement, discomfort, unhappiness, and conflict with family were suggested by an elevation on Scale 4 (Pd; T = 72), and tension, anxiety, rumination, and sleep disturbances were suggested by an elevation on Scale 7 (Pt; T = 68). Elevation on supplemental scales suggested that he may have been passive and submissive, slow and painstaking, conventional, was experiencing sleep disturbance, was feeling like a failure, and was resentful but did not know how to express his angry feelings. His content scale scores showed elevation in bizarre mentation indicative of hallucinations and delusions - symptoms which were interfering with work and occupational tasks. His content scale scores also reflected insecurity, a tendency to give up easily in the face of hardship, and views that he could not be understood. This patient also showed elevation on clinical scales measuring introversion ((Si; T = 76) and (INTR; T = 66)), social discomfort (SOD; T = 84), and tendencies to internalize and have a careful and cautious lifestyle (R; T = 72). On the TAT, the patient’s stories were simplistic, concrete, and brief. His stories included themes of feeling frustrated about current circumstances but being hopeful for the future, feelings of confusion, themes of abandonment, being left and not paid attention to, and feelings of regret and shame. His answers were vague throughout the test and he needed frequent prompting. In response to a blank card (16), he was unable to create a cohesive story or identify any specific characters who had thoughts or emotions, suggesting that he had a difficult time organizing his thoughts and emotional content in unstructured environments. The test results did not reveal formal thought disorder. The consulting psychologist did not find evidence of schizophrenia in the test results. His test results suggested a possible mood disorder such as unspecified bipolar disorder in a likely high functioning patient with ASD.

The patient improved and was discharged on Lurasidone 40mg daily, Benztropine 1mg twice daily, Quetiapine 50mg nightly, Fluoxetine 20mg daily, Losartan 50mg twice daily, and Clonidine 0.2mg nightly.

## Discussion

The complexity of this case lies in the overlap of symptoms between ASD and SSD. Both conditions have similarities in the deficit of interpersonal interactions, bizarre ideas, interpersonal sensitivity, etc.; however, SSD is a progressive condition that leads to functional deficit, chronic maladjustment, and requires continuous antipsychotic treatment [3]. Individuals with high functioning ASD can be very well adjusted in life and have minimal functioning deficit. Our patient was able to achieve college degrees, sustain occupations, and live independently. The core deficit in ASD is failure to mentalize, which leads to misinterpretation of social cues. This leads to erroneous ideas about the intentions of others when under stress. This is what our patient demonstrated. His defenses included dissociation (episodes of amnesia) and rationalization (he understood his hallucinations were not real). Mature defenses included suppression (he tried his best to put his thoughts and hallucinations aside so he could focus on his occupation). The fact that he understood his hallucinations were not based in reality was further proof that he did not fit the diagnosis of schizoaffective disorder as well as previously thought and perhaps fit the diagnosis of ASD better.

The patient is genetically vulnerable to psychiatric disorders given his family history of a mental health diagnosis in his brother and gambling addiction in his father. Studies have found that ASD may be three to four times more prevalent in males than females [4]. Medication nonadherence, sleep deprivation, lack of proper treatment, and lack of proper follow up had precipitated these factors. He had some difficulty with social understanding and isolated himself secondary to social anxiety. Childhood bullying and accusations of homosexuality created a conflict between identity versus confusion in the patient. This unaddressed relationship conflict and occupational stress may have provoked the development of psychotic experiences. He had been avoidantly attached, had low self-esteem, had the development of a psychodynamic internal unconscious conflict and defense, and had an interpersonal chronic unresolved conflict. He had internalized ideas of homosexuality. He had been unable to maintain consistent interpersonal relationships due to being avoidant since the incident in his childhood.

Aside from sleep difficulty, he did have good overall health, a positive response to low doses of medication, was of above-average intelligence, had never used substances, and was reflective of and could modulate his affect. He previously responded well to psychotherapy, had good insight, and had a positive relationship with his parents. 

ASD is currently diagnosed based on behavior; however, not all patients present meeting all the criteria. Assessment criteria should be clarified to better understand the effects that ASD has across the lifespan. An ASD diagnosis requires that symptoms begin in childhood; however, milder cases of ASD may not fully present until adulthood when the demands of social life, maintaining an occupation, etc. increase. Retrospective reviews have found that ASD in adults may present with many comorbid mental health disorders including anxiety, depression, OCD, psychosis, bipolar affective disorder, and self-harm. Additionally, comorbid gastrointestinal disorders, sleep disorders, hypertension, hyperlipidemia, cerebrovascular conditions, immune conditions, obesity, seizures, and Parkinson’s disease are prevalent. It has been proposed that abnormal melatonin production or circadian timing contribute to sleep disorders in ASD patients. Anxiety, especially social anxiety, is particularly prominent among adults with ASD. Studies have found that OCD in ASD patients presents with sexual obsessions most often while OCD in patients without ASD often presents with somatic obsessions [5]. 

The patient provided an account of recurrent, impulsive behavior throughout his life. This could demonstrate impulsivity in the context of a mood condition as well as poor planning and social reciprocity in the context of ASD.

Psychological testing can provide valuable insight when a psychological diagnosis is uncertain. In differentiating between SSD and ASD, the MMPI has proven to be very specific. Those with ASD consistently score higher on clinical scale Si, personality psychopathology five (PSY-5) scale introversion (INTR), content scale social discomfort (SOD), and supplementary scale repression (R) [6].

Psychotic experiences not caused by psychotic disorders including hallucinations, delusions, and flattened affect have been found to exist among the common population at rates of 5%-12%. A study found that having ASD increases the risk of developing psychotic experiences by two to five times. Many studies suggest that ASD may foresee the development of psychotic experiences. Schizophrenia is also far more common among ASD patients when compared to the general population. Negative childhood experiences such as bullying may foster the development of schizophrenia in ASD. The two diagnoses may also be confused. The issues with social communication as well as difficulties in understanding the intentions of others that are characteristic of ASD foster the development of delusions [7]. 

## Conclusions

In conclusion, the case presented here demonstrates how ASD can present atypically; however, the patient’s diagnosis remains dynamic and future hospitalizations can continue to explore the diagnosis. Diagnostic criteria for ASD and SSD often overlap. Determining the primary disorder is key to appropriate treatment. By investigating personality traits and defenses, psychological testing often leads to diagnosis. This case was a diagnostic conundrum in which psychological testing and thorough interview revealed that the patient does not have a schizoaffective condition but rather, high functioning ASD with brief psychosis when under stress. The fact that his report of delusions and hallucinations while he was calm and composed on the unit got better on a low, non-antipsychotic dose of Lurasidone supports this theory. Treatment should be based on the primary disorder and most relevant disturbance.
